# Healthism and Digital Self‐Tracking: Reinventing the Individualistic Ethos in Chile

**DOI:** 10.1111/1467-9566.70097

**Published:** 2025-10-08

**Authors:** Rodrigo De La Fabián, Alvaro Jiménez‐Molina, Francisco Pizarro Obaid, Jimena Carrasco Madariaga

**Affiliations:** ^1^ Faculty of Psychology Universidad Diego Portales Santiago Chile; ^2^ Faculty of Psychology and Humanities Universidad San Sebastían Santiago Chile; ^3^ Faculty of Medicine Universidad Austral de Chile Valdivia Chile

**Keywords:** Chile, digital self‐tracking, fitness apps, healthism, insulin pumps, posthumanism, quantified self, science & technology studies

## Abstract

This article examines how digital self‐tracking practices (DSTPs) reconfigure the individualistic ethos of healthism through a posthumanist onto‐epistemological shift. Healthism, originally formulated by Robert Crawford in 1980, refers to an ideology that prioritises individual health as the foundation of well‐being, promoting self‐management through lifestyle choices and placing responsibility on individuals. Based on 23 interviews with users of insulin pumps and fitness applications in Chile, this study employs a critical thematic analysis informed by Science and Technology Studies. Findings show that DSTPs reproduce and transform the logic of healthism, reinforcing responsibility while reshaping the conditions under which health is known and managed. Insulin pumps embed users in tightly regulated medical regimes, whereas fitness applications foster gamified self‐optimisation and promote competition. Both technologies intensify the individualistic ethos while displacing introspection with data‐driven knowledge. This highlights a crucial paradox: DSTPs legitimise themselves through discourses that deny the very power of discourse, elevating numbers as the only credible source of truth. Thus, the digital subject is caught between the demand to ‘know thyself’ and the recognition that such knowledge depends on technologies that both empower and constrain it.

## 1 Introduction

Crawford (1980) coined the term ‘healthism’ to describe an ideology that prioritises personal health through lifestyle choices, merging medical and therapeutic cultures (Nehring et al. 2020). Rooted in holistic and self‐help movements, it promotes individual responsibility for health, framing behaviours and emotions as targets for intervention. While appearing to challenge traditional medicine, healthism ultimately reinforces medicalisation, with popular psychology offering health strategies beyond clinical settings. Crawford (1980) argues that this focus on the individual depoliticises public health by undermining collective approaches, fostering the illusion of personal control while neglecting broader social determinants.

Since Crawford's analysis, scholars have demonstrated how neoliberalism and entrepreneurial subjectivity have intensified the emphasis on individual responsibility for health (Schrecker and Bambra 2015; Schrecker 2016; Aamann 2020; Spratt 2023; Card and Hepburn 2023). People are encouraged to self‐manage, self‐regulate and assess risks, shifting from the right to health to the duty to stay well (Greco 1993; Mattioni et al. 2021). This transformation produces a new form of ‘patienthood’ in which, as Rose (2007, 23) observes, individuals are no longer passive recipients of care but active consumers who demand information, expect results and hold providers accountable, thereby reshaping the relationship between health, subjectivity and responsibility.

In recent years, the rapid proliferation of portable digital devices has enabled unprecedented possibilities for self‐management of health and healthy lifestyles (Lupton 2016; Insel 2017; Sanders 2017; Ajana et al. 2022). Equipped with sensors that generate continuous, real‐time data, these technologies have given rise to digital self‐tracking practices (DSTPs), providing a quantified wisdom of the self (Wolf 2009, 2010). Digital self‐tracking practices (DSTPs) are sociotechnical practices of self‐monitoring mediated by digital technologies—such as wearables (e.g., smartwatches, fitness trackers), mobile applications and biosensors—that allow the real‐time recording and tracking of physiological and behavioural data. These practices encompass indicators such as heart rate, step count, caloric intake, sleep quality, mood and blood sugar levels and are typically oriented towards objectives related to health, physical performance and subjective well‐being.

Although some researchers view these new technologies as a means of empowering individuals to manage their own health (Swan 2012; Topol 2015), others approach them from a more critical perspective. From this standpoint, the transfer of responsibility for health from the state to the individual, coupled with an emphasis on personal choice over structural determinants, is closely tied to the neoliberalisation of Western societies (Lupton 2013a, 2013b; Boka and Pöll 2016; Elias and Gill 2018; Badr 2022; Wieczorek and Rossmaier 2023). These practices are also interpreted as drawing on ‘psy’ discourses, fostering what Lupton (2016, 67–68) terms a ‘reflexive monitoring self’ and legitimising individualised approaches to health management. Within this framework, DSTPs are understood as *technologies of the self* (Foucault 1988) that construct the subject as a self‐designed project (Yilmaz and Mermutlu 2023, 54), embodying the ideal of the ‘neoliberal entrepreneurial citizen’ (Lupton 2016, 68).

However, the possibility of directly linking healthism with DSTPs through neoliberalism requires critical reflection. While healthism often involves forms of mediation—such as therapy or self‐help literature—these still presuppose a subject capable of interpreting and transforming itself through reflective understanding. In contrast, DSTPs assume that a direct self‐to‐self relationship is impossible and must be outsourced to the technological (De Vos 2020). As Wolf (2010) suggests, their core assumption is that human self‐knowledge is fundamentally flawed: We cannot fully register or process the details of our daily lives. This anthropological diagnosis legitimises self‐tracking as a necessary means of accessing personal truth. At the same time, it signals a shift in the epistemology of the self—from one grounded in introspection and discursive elaboration to one increasingly shaped by data‐driven, technologically mediated knowledge.

This article hypothesises that DSTPs reinforce and transform neoliberal subjectivity and the normative pursuit of healthy lifestyles, revealing key continuities and discontinuities with healthism. We approach DSTPs as sociotechnical assemblages comprising multiple human and nonhuman agencies—such as users, technologies and both lay and expert knowledge—embedded within broader discourses and practices concerning the body, technology and subjectivity (Latour 1983, 2005; Pickering 1995; Lupton 2020). These assemblages support a dynamic interplay between emerging modes of subjectivation and the digital production of self‐truth.

To empirically analyse how these assemblages operate, this article draws on two Chilean case studies: the use of insulin pumps (IPs) and fitness applications (FAs). IPs are digital medical devices that monitor glucose levels in real time through interstitial fluid in individuals with diabetes, enabling the continuous and automated delivery of insulin. In contrast, FAs rely on everyday devices such as smartphones and smartwatches to track aspects of health and well‐being, including sleep, mood and physical performance. These practices correspond to two of the five types of DSTPs identified by Lupton (2014b), and together, they allow for a comprehensive analysis of voluntary self‐tracking.^1^ IP use aligns with ‘pushed self‐tracking’: practices aimed at managing or treating diseases and health conditions, where patients remain active decision‐makers while adhering to medical standards (Lupton 2013c, 2014b; Erikainen et al. 2019; U.S. Food and Drug Administration 2019; Lee et al. 2023). In contrast, FAs correspond to Lupton's (2014) category of ‘private self‐tracking’—also referred to by the FDA as low‐risk general wellness products (U.S. Food and Drug Administration 2019).

Chile offers a significant context for examining the evolution of healthism. As one of the first countries to adopt neoliberalism (Harvey 2007; Klein 2007), it pioneered the privatisation of healthcare in Latin America in 1979 (Bruce 2000). The resulting dual system of private (ISAPRE) and public (FONASA) insurers shifted responsibility for health management from the state to individuals, who must navigate a market‐oriented environment with limited institutional support (Unger et al. 2008). Access to care depends not only on income but also on the ability to actively manage coverage and risk, reinforcing long‐standing inequalities (Rotarou and Sakellariou 2017). In this context, health is framed as a moral obligation and personal responsibility, obscuring structural determinants through discourses of self‐care and lifestyle optimisation. This configuration produces what Araujo and Martuccelli (2012) term the ‘hyper‐actor’: An individual held intensely accountable for their destiny and forced to manage life amid institutional fragility and pervasive inequality. Unlike the liberal ideal of autonomy supported by collective guarantees, the hyper‐actor embodies an unsupported agency in which risk and well‐being are privatised and life is experienced as a series of continuous tests.

Additionally, Chile stands out as a regional leader in adopting portable digital technologies (Universidad San Sebastián [USS] 2018; GSMA 2024) and digital health initiatives (Ministerio de la Salud [MINSAL] Forthcoming; Rojas et al. 2019; Bianchi et al. 2023). Moreover, since 2017, Law No. 20.850—known as the ‘Ricarte Soto Law’—has fully covered the cost of state‐of‐the‐art insulin pumps^2^ and related supplies for eligible patients with Type 1 diabetes mellitus who meet specific severity criteria, regardless of their socioeconomic status (Superintendencia de Salud Forthcoming). This combination of neoliberal health governance and expanding access to advanced tracking technologies provides fertile ground for investigating how intersecting regimes of responsibilisation, technology and care shape voluntary digital self‐tracking practices.

This article concludes that DSTPs reconfigure healthism by integrating their individualist ethos with posthumanist frameworks of subjectivation. In this new regime, individuals are held responsible for managing a molecularised body accessible only through digital devices, placing them in a paradoxical position of intensified autonomy and growing dependence on algorithmic and data‐driven forms of knowledge. Although DSTPs reproduce healthism's individualistic and responsibilising ideology, they also introduce a new digital metric of self‐knowledge, enabling novel modes of subjectivation that both extend and transform the neoliberal framework of self‐care.

## 2 Materials and Methods

This article is part of the research project ‘Análisis de los Procesos de Subjetivación en Prácticas de Automonitoreo Digital en Chile: los casos del uso de bombas de insulina y las aplicaciones fitness’—‘Analysis of Subjectivation Processes in Digital Self‐Tracking Practices in Chile: The Cases of Insulin Pump Use and Fitness Apps’—(De La Fabián et al. 2024). The study aims to investigate the impact of digital self‐tracking practices on social life and contemporary understandings of the self. To this end, it focuses on two empirical cases: the use of insulin pumps by individuals diagnosed with Type 1 diabetes mellitus and the use of fitness applications. It unfolds in two main phases. Phase 1 involves semi‐structured interviews with users of insulin pumps (IPs) and fitness applications (FAs); Phase 2 consists of ethnographic fieldwork. The present article draws on the results of Phase 1.

### 2.1 Participants

Interviewees were recruited primarily through flyers circulated on social media platforms and, secondarily, via snowball sampling. In all cases, participants initiated contact voluntarily to safeguard their autonomy and privacy. IP and FA users were stratified into two socioeconomic categories: upper‐middle status (UMS) and lower‐middle status (LMS).

For the insulin IP group, inclusion criteria included being 18 years of age or older, having a diagnosis of Type 1 diabetes mellitus and having used digital self‐monitoring devices to manage the condition for at least 1 year. As shown in Table 1, 12 individuals were interviewed—10 women and two men—aged 18 to 52. Of these, seven were classified as UMS and five as LMS. Except for one participant who used only a continuous glucose monitor (FreeStyle Libre 1), all others were enroled in the Ricarte Soto Law programme and used one of two Medtronic models: the MiniMed 670 g or the MiniMed 780 g.^3^

**TABLE 1:** Insulin pump sample.

	Nickname^a^	Age	Gender	Socioeconomic status^b^	Self‐tracking technology
1	Cecilia	22	F	UMS	MiniMed 670g
2	Sofía	18	F	LMS	MiniMed 780g
3	María	33	F	UMS	MiniMed 780g
4	Susana	25	F	LMS	MiniMed 670g
5	Isabel	51	F	LMS	MiniMed 780g
6	Pablo	26	M	UMS	MiniMed 670g
7	Juan	35	M	UMS	FreeStyle Libre 1
8	Clara	25	F	UMS	MiniMed 780g
9	Elena	26	F	UMS	MiniMed 780g
10	Julia	27	F	LMS	MiniMed 780g
11	Ema	37	F	UMS	MiniMed 670g
12	Lucía	52	F	LMS	MiniMed 670g

^a^ The participants' names have been altered to protect their anonymity and safeguard the confidentiality of the information.

^b^ To categorise participants' socioeconomic status (SES), the following criteria were considered: (1) commune of residence, as a territorial indicator associated with SES stratification in Chile; (2) highest educational level completed; (3) current occupation; (4) type of educational institution for those still studying (e.g., public, subsidised or private school, university, technical institute); (5) source of funding for higher education (e.g., private, state‐guaranteed loan [CAE], public grant); (6) health insurance system affiliation (public system—FONASA—or private insurers—ISAPRE).

For the FA group, inclusion criteria required participants to be 18 or older and to have used fitness applications regularly for at least one year. Given the diversity in how fitness applications are employed, participants were categorised according to the scope and focus of their self‐tracking practices. The first category, ‘focused users’ (FUs), included individuals who engaged with one or a few applications for specific monitoring purposes—such as tracking running performance or dietary intake. The second category, termed ‘integrated users’ (IUs), comprised those who used multiple applications across various domains, often without a singular or consistent focus.

As shown in Table 2, 11 individuals were interviewed—three women and eight men—aged 22 to 43. Of these, eight were classified as upper‐middle status (UMS) and three as lower‐middle status (LMS). All participants used both smartphones and smartwatches, relying not only on built‐in tracking features (e.g., heart rate, sleep patterns) but also on third‐party platforms such as Strava and FatSecret. Based on their usage patterns, six participants were classified as focused users (FUs), whereas the remaining five were identified as integrated users (IUs). This typology was developed to reflect variations in the intensity and scope of digital self‐tracking, allowing for a more nuanced understanding of how these practices are embedded in users' everyday lives.

**TABLE 2:** Fitness application sample.

	Nickname	Age	Gender	Socioeconomic status	User category
1	Lucas	43	M	UMS	FU
2	Leo	22	M	UMS	FU
3	Olivia	41	F	UMS	IU
4	Mateo	31	M	LMS	FU
5	Gabriel	26	M	UMS	FU
6	Adrián	43	M	UMS	IU
7	Daniel	26	M	UMS	FU
8	Ana	24	F	UMS	IU
9	Nicolás	23	M	LMS	FU
10	Victoria	25	F	LMS	IU
11	Tomás	26	M	UMS	IU

### 2.2 Analytical Approach

The qualitative analysis of the interviews was conducted using a critical thematic analysis approach (Braun and Clarke 2021). Rather than merely identifying patterns in the data, this approach served as an interpretative framework, aligning with the reflexive thematic analysis tradition (Fereday and Muir‐Cochrane 2006). Central to this perspective is recognising that themes do not simply ‘emerge’ spontaneously from empirical data. Instead, the researchers actively construct themes through interpretative engagement with theoretical frameworks, research objectives and situated conceptual understandings. Unlike more positivist methods, this approach explicitly acknowledges the interpretative nature of coding, emphasising the dynamic interplay between theory and empirical observations (Collins and Stockton 2018). Consequently, the analysis was designed as an iterative and reflexive process characterised by continual dialogue between the data and theoretical insights.

The analysis was conducted in three interconnected stages. First, the four authors collaboratively reviewed a subset of 10 interviews—five from insulin pump users and five from fitness application users—to identify initial units of meaning and develop a shared interpretative framework. This initial phase involved open coding coupled with collective discussions to refine conceptual understanding and analytic consistency. In the second stage, we developed a flexible coding scheme that combined emergent, inductively derived categories with theoretically informed ones. Two researchers applied this coding scheme to the 13 remaining interviews. This hybrid coding approach (Fereday and Muir‐Cochrane 2006; Braun and Clarke 2021) enabled us to strike a balance between openness to empirical detail and conceptual rigour, continually refining codes through iterative revision to ensure consistency and relevance. In the third and final stage, the research team synthesised the identified codes into central themes—such as bodily knowledge, forms of subjectivation, health self‐management, gamification and competition, routinisation and standardisation, digital data saturation and technological ambivalence, among others. Through this thematic synthesis, we aimed to explore and elucidate the material, affective and normative implications of DSTPs on participants' everyday lives, explicitly connecting empirical findings with broader theoretical frameworks.

### 2.3 Ethical Considerations

The Research Ethics Committee of Universidad Diego Portales (Santiago, Chile) reviewed and approved the research protocol (Approval No. 17‐2024, 23 May 2024). All participants signed an informed consent form authorising the use of their anonymised interviews for scientific purposes. The research team took all necessary measures to ensure confidentiality and protect the participants' identities.

## 3 From Intuition to Data: Becoming a Digital Self‐Tracker

The meaningfulness and legitimacy of digital self‐tracking practices (DSTPs), such as insulin pumps (IPs) and fitness applications (FAs), do not arise intrinsically from the devices themselves. Instead, they depend on users cultivating specific ways of making sense of their bodies and technologies that often diverge from the assumptions underpinning healthism.

As Crawford (1980, 371) acknowledges, his perspective is grounded in a molar conception of the body (Rose 2007). This conception derives from the anatomopathological model of the late eighteenth century, in which the body was considered fully accessible to experience and knowable through the observable manifestation of signs and symptoms (Foucault 1991, 95). As a result, the body's truth is presumed to be reachable through introspection and the clinical gaze, thus aligning with a Cartesian‐humanist epistemology (Dennett 1991, 67).

By contrast, DSTPs rely on biosensing technologies that render previously imperceptible physiological and micro‐behavioural processes legible through data (Nafus 2016; Bidargaddi et al. 2016; Onnela and Rauch 2016). These technologies introduce an ontological and epistemological shift, as they no longer operate on the molar body but on the molecular (Rose 2007).

According to Rose (2007, 12), the emergence of the molecular body is closely tied to advances in biotechnology that shift attention from visible structures to a finer scale, where life is understood in terms such as coding sequences and gene expression. Our definition emphasises that, at the molecular level, direct experience is no longer possible: Both the contemporary clinician and the user of DSTPs must rely on technological mediation to access and interpret processes that would otherwise elude them. Unlike the perceptible molar body, which can still be approached through discursive and phenomenological frameworks, the molecular body is infra‐subjective and asignifying (Lazzarato 2006, 2014). Thus, it is not apprehended through language but mostly through numerical matrices—graphs, statistical models and algorithmic visualisations—that render it intelligible. DSTPs open access to this new scale of the self, aligning with posthumanist and behaviourist perspectives that promote the ‘laboratorisation’ of everyday life and cultivate a growing distrust in human capacities for self‐knowledge (De La Fabián et al. 2023; De La Fabián 2024).

In the case of IP use, this shift in understanding and perceiving the body does not begin with the device itself but with the initial diabetes diagnosis—a moment that interviewees describe as a *‘major shock’* (Juan; Julia) or *‘a difficult moment’* (María), marking a rupture in their biographical trajectories. From that point on, and before adopting an insulin pump, individuals embark on a lengthy learning process, acquiring a highly technical language and knowledge, as well as skills for managing their health condition, often guided by healthcare professionals and peers.

Notwithstanding, this learning process is not just about managing diabetes but about adapting to a new way of relating to the body at a molecular level. This dimension is not immediately perceptible and can only be accessed through technologies such as finger‐pricking devices or continuous glucose monitors (CGMs).^4^ In this context, people with diabetes learn to trust technological devices while distancing themselves from the perceptible signs of their bodies. For example, Susana, recalling how she managed her diabetes before having a CGM, states the following:

I was asymptomatic. So, for instance, I wouldn’t realise when my blood sugar was really low. I only noticed when it was already critical.

The knowledge required to manage the illness at a molecular scale is primarily numerical rather than discursive. This transformation is particularly evident in individuals who previously relied on bodily sensations and discursive reasoning to manage their diabetes. For instance, Julia, who moved from another Latin American country to Chile, describes how her understanding and management of diabetes evolved:

[Before arriving in Chile and enrolling in the diabetes programme,] I didn’t know what range was, I didn’t know what target blood sugar was, I didn’t know what postprandial blood sugar was, things like that. […] I would just guess [‘*al ojo*’] before eating.

In contrast, to qualify for an insulin pump in Chile, she had to acquire a different form of knowledge:

That’s when the marathon begins because you need approval from a nutritionist, who must certify that you know how to count carbohydrates, manage hypoglycemia, and handle hyperglycemia.

(Julia)

The interviewee's experience highlights the transition to IP use, which involves a learning process grounded in a new epistemology, where truth is established through quantifiable data, such as carbohydrate counting and statistical ranges.

On the other hand, FAs also operate at a molecular scale. Variables such as heart rate, sleeping patterns or step count—continuously measured in natural and everyday environments—can be just as opaque to our direct understanding as blood sugar levels.

[When I go for a run without my smartwatch] since I already know my body and what it means when I feel a certain way […] I think I have an idea of my pace. But I’m still left wondering, like, in more detail, how exactly I ran, [because] the app usually tells me, and the numbers are super precise. For example, I usually run at a pace of [5 minutes 30 seconds per kilometre]. So, let’s say one day I’m feeling good, and I manage 5:25—man, the difference is huge. […] So yeah, you kinda get that itch to know exactly how you did. And then you realise, damn, you lost that data, and it’s a bit frustrating.

(Lucas)

In this vignette, the same two types of knowledge we found in Julia can be observed. The first is molar‐intuitive, as reflected in the interviewee's statement that he knows his body well enough to determine his running pace without needing a smartwatch. The second involves infra‐subjective knowledge and follows a numeric rather than discursive framework, evident in the five‐second difference only a digital device can detect.

However, unlike the case of IP, becoming an FA user does not entail a significant rupture in one's life trajectory or rely on expert knowledge or formal institutions. Instead, this subjective transformation is shaped by popular cultures surrounding digital technology, the body, consumption practices, sports and healthy lifestyle trends. This onto‐epistemological assumption becomes evident in the following accounts, which explicitly suggest that FAs provide access to what we *truly are* and *do*, implicitly contrasting this with how we perceive ourselves.

So yeah, what the app helps with is knowing whether you’re *actually* in an energy deficit or not.

(Leo)

Honestly, I started using it [a smartwatch] to check the numbers, to see if the exercise *really* works for me or not.

(Victoria)

The self is no longer trusted as the primary source of knowledge. Instead, the truth about the body is deferred to data produced by digital devices operating below the threshold of perception. What counts as *real* is not what is felt or narrated but what is measured. However, in the interviews, no explicit references exist to how FA users acquire this onto‐epistemological frame. It unfolds imperceptibly through routine interactions with applications and devices, naturalising a mode of subjectivation in which individuals come to know and govern themselves according to data metrics and algorithmic feedback—a quiet yet profound transformation in the contemporary experience of the self.

In sum, although IP use originates in a medical rupture and requires expert‐led instruction, FA use emerges informally through everyday health routines and self‐optimisation trends. Despite these distinct pathways, both cases illustrate a progressive shift towards modes of self‐relation grounded in data interpretation rather than embodied sensation. This transformation is not solely cognitive or behavioural; it reconfigures the very ways in which the body is perceived, trusted and governed. Unlike the experiential and discursive orientation of healthism, DSTPs promote a mode of subjectivation rooted in technological mediation and numerical rationality.

## 4 Autonomy, Responsibility and the Paradox of Control

Crawford's (1980) concept of healthism frames health management as a matter of individual moral responsibility, privileging self‐discipline and bodily surveillance. In general terms, DSTPs—such as IPs and FAs—share this individualising ethos. They promote practices centred on autonomy and reinforce the idea that managing one's health is ultimately a personal responsibility, grounded in self‐care rather than dependence on others.

Consequently, IPs and FAs present themselves as seamlessly integrated into everyday life, unobtrusive, adaptable and responsive to individual needs. For instance, FAs enable users to train in various settings, thereby reinforcing ideals of autonomy and self‐direction. Lucas likens his smartwatch to a virtual coach that tells him ‘everything he has to do,’ illustrating how it supports his independence by replacing the need for a physical trainer. Likewise, IPs reduce dependence on regular medical supervision or rigid meal planning, allowing users to maintain daily routines with fewer disruptions and reinforcing the notion that effective health management should be discreet, personalised and self‐directed.

I truly feel more confident exercising, knowing that I have, so to speak, someone monitoring me and alerting me if something goes wrong.

(Susana)

However, the promise that digital devices would seamlessly integrate into our lives as mere supplements whose sole or primary function is to enhance our freedom and autonomy remains more of a techno‐utopia than a reality (Chrysanthou 2002). Such narratives conceal the extent to which these technologies fail to liberate users from various tasks, redefine their roles and reshape the lives they claim to support.

In the case of IPs, users quickly come to realise the gap between this idealised vision and their lived experience, as these devices often demand constant monitoring, adjustment and interaction.

I would tell [the healthcare staff] not to lie; please just don’t lie. I honestly felt completely deceived because they truly promised me that this was going to be amazing—that I wouldn’t have any problems, that I would get better overnight, that my glycated haemoglobin would be fine, that my health would be perfect, that I would never have hypoglycemia again. They promised me so much, and it just wasn’t true.

(Elena)

The expectation was that I would have this amazing freedom, that the pump would do everything for me because I was told that the pump was an artificial pancreas […] I basically thought it was a magic machine.

(Sofía)

These testimonies highlight the dissonance between the optimistic discourse surrounding IPs—specifically, the ‘artificial pancreas’—and the everyday reality of their use. Far from granting effortless autonomy, the devices often reconfigure users' daily routines and self‐relations, demanding vigilance and labour. What emerges is not a seamless integration but a subtle redistribution of responsibility.

This strained relationship with healthcare professionals extends beyond the initial disappointment. Users often feel that their daily experiences are not fully understood, which can sometimes lead them to question or even contradict medical advice.

My worst days are the ones when I’m on shift obviously, because my cortisol levels are through the roof. So, the nurse tells me, ‘Don’t mess with it’ [‘*no le metas mano*’], but I just can’t. So, I keep adjusting it, and it works for me.

(María)

Hence, expert knowledge does not relieve the burden of responsibility for managing diabetes. Faced with the impression that healthcare professionals lack a complete understanding of the lived reality of their condition, users often feel compelled to rely on their own experience and knowledge, sometimes at the expense of medical expertise.

In the same vein, and contrary to expectations, many interviewees report that IPs do not ease the demands of health self‐management; instead, they amplify them, reinforcing the imperative of continuous self‐monitoring.

At first, though, I thought it would take some of the burden off me, that I’d be a bit less tied to constantly managing my diabetes. But no, I actually think having a pump requires even more responsibility.

(Sofía)

IPs require continuous user engagement, involving routine tasks such as calibrating the GSM by pricking the user's finger to measure blood glucose, counting carbohydrates and adjusting for physical activity. Beyond these demands, users report being constantly vigilant due to concerns such as the risk of damaging the device, frequent alarms and potential inaccuracies in glucose readings or malfunctions.

Honestly, I think our days are always 24 hours long. We don’t really get much rest. People who have been doing this for longer and have achieved stability do get more rest. But we never fully rest, not 100%.

(Susana)

Thus, interviewees provide various examples illustrating that they cannot blindly follow the instructions given by the IP. In other words, they must constantly monitor the system that monitors them. In this respect, an interviewee explained that when she had to change the glucose sensor, which typically occurs once a week, during the first days, she had to prick her finger and measure her blood glucose multiple times. This process of calibration takes a few days, during which:

At night, my levels are always steady—80, 90—but the pump shows, I don’t know, below 50, and I know that’s not true. [So], I ignore it. And I know that’s not the right thing to do, but sometimes the pump goes off at 3 a.m., and I’m exhausted, and I don’t feel bad. No. I just say, ‘Okay,’ and go back to sleep. Now, if it goes off again, then I’ll check. But if I don’t believe it, I just ignore it.

(Sofía)

Therefore, on the one hand, IPs automate processes and help outsource the burden of health self‐management. However, at the same time, users remain the ultimate judges of the accuracy of the information generated by these devices. This dual stance of trust and doubt, of relaxation and vigilance, is clearly expressed in the following vignette:

The truth is, yes, I trust the pump a lot, but I’m still always aware […] it still relies on my human factor, and I’m not that great at it, to be honest.

(Isabel)

Although IPs assist with monitoring and regulating glucose levels, they do not replace the need for continuous decision‐making and self‐discipline. They enable a form of over‐responsible subject, who has to constantly interpret data, adjust their behaviours and ensure the system functions correctly. This places users in a complex and paradoxical position. The ultimate truth of diabetes management lies in its molecular nature and can only be accessed through advanced technology. Yet, even without direct access to this truth, they bear full responsibility for decision‐making. They must continuously interpret—and sometimes override—the device, making critical choices based on incomplete or ambiguous information.

This complex and situated position of IP users challenges Conrad's (1992) distinction between health concerns mediated by medicine and those rooted in self‐regulation and lifestyle. From his perspective, healthism is external to medicine, oriented towards nonclinical interventions. However, as Turrini (2015) notes, such boundaries often dissolve in practice. Users of IPs act as *bricoleurs*, drawing simultaneously on medical expertise, technological information and their own lived experience. Their management of diabetes exemplifies how responsibilities and interventions are hybridised, demanding a more nuanced understanding of how health is enacted in everyday life.

Notwithstanding, although IPs and healthism emphasise self‐responsibility, they do so in markedly different ways. Healthism involves specific forms of mediation—such as therapists or self‐help literature—that grant individuals greater flexibility in defining the terms of their self‐care. In contrast, the use of IPs is not merely supported by expert knowledge and data‐driven monitoring; it is shaped and sustained by them. This means that, for IP users, responsibilisation does not imply independence from authority but rather redefines dependence as a constitutive condition of self‐management. In other words, as in healthism, users ultimately remain responsible for managing their health. However, their position is more precarious, as they are often compelled to rely on forms of knowledge that are culturally devalued, such as intuition and lived experience. Asserting this knowledge, therefore, entails a form of epistemic disobedience, which intensifies the sense of solitude inherent in the practice of self‐care.

## 5 Competition and the Data‐Driven Standardisation of Everyday Life

Unlike the medicalised framework surrounding IP use, the onto‐epistemological orientation of FAs is typically adopted through mainstream cultural channels shaped by wellness discourses and prevailing social norms. The concept of *healthism* has been particularly insightful in revealing the growing role of lifestyle as a means of individual self‐expression, manifested in consumption habits, leisure activities, clothing and bodily dispositions (Featherstone 1987). In the context of health promotion and illness prevention, personal choices around exercise and diet have gained increasing relevance, further embedding FAs within the cultural logic of healthism.

However, important distinctions remain. Although Crawford's (1980) notion of healthism emphasises introspective, discursive forms of self‐understanding, FAs promote a data‐driven rationality rooted in numerical metrics, performance tracking, behavioural optimisation and competition.

This shift must be understood within the broader historical context of the expansion of quantification. Although statistical reasoning has long underpinned institutional governance (Hacking 1990; Miller 2001), its extension into the intimate sphere of everyday life has intensified with the rise of neoliberal audit cultures (Shore and Wright 2018). Originally designed to evaluate institutional performance, audit rationalities have been internalised by individuals, who increasingly assess themselves through standardised metrics and continuous comparison.

FAs amplify this dynamic by embedding users in regimes of measurement, perpetual self‐improvement and accountability. As a result, success or failure in health and wellness management is framed less as a matter of social or structural conditions and more as a reflection of individual achievement or shortcoming.

This logic is evident in how users describe their experience with tracking platforms such as Strava:

Strava always compares you to yourself. When you run your fastest 10K or your fastest kilometre within a run, it notifies you. It gives you something like *‘Today’s achievements'* and even a little medal. It tells you, *‘On this run, you ran your fastest two‐km segment ever.’* […] And it also notifies you about comparisons with others—for example, if you ran a segment that other people have completed before.

(Lucas)

By enabling the real‐time collection and analysis of biometric data at both the intraindividual (*N* = 1) and interindividual (*N* = all) levels (Schork 2015; Onnela 2021), FAs facilitate not only self‐quantification but also the integration of personal data into broader information systems. In doing so, they actively position the user within a wider landscape of collective metrics, fostering a continuous comparison and performance evaluation regime. Unlike IP use or healthism—both of which are primarily oriented towards intraindividual regulation—FAs introduce a horizontal axis of competitive visibility. This dynamic reconfigures health self‐management as not merely a private responsibility but a publicly legible activity, subject to ranking and comparison. To achieve this, FAs utilise the rationality and aesthetics of gamification, where game‐like elements enhance engagement by transforming self‐tracking into a structured challenge with rewards and progress markers (Arora and Razavian 2021).

Once introduced, competition becomes an integral part of the sporting experience, shaping motivation and self‐perception.

At first, I was competing—I would go out running just to finish first, and that alone made me feel bad. I thought, ‘*This is stupid.’* Then my teammate, who was in second place, told me the same thing—that he was running just to get first place. So even today, sometimes I catch myself checking, ‘*Am I in the first place? Am I in second?’* That kind of competition, I actually find it good.

(Tomás)

What starts as a simple physical activity is reframed through rankings and performance comparisons, fostering a continuous drive for improvement. Even when users recognise the pressures of competition, they often see it as a positive force, reinforcing engagement and commitment to their routines.

In the same vein, the following FA user differentiates between sports activities that he considers crucial to track—such as running—and those that should be ‘protected’ from measurement and the competitive ethos:

Tennis, on the other hand, not at all—for me, it’s something I enjoy, it’s playful, it’s my hobby […] I would protect it. I don’t want to tangle tennis, my sport, with too much tracking and data, no.

(Lucas)

This distinction reveals that self‐tracking not only registers and analyses performances but also alters their meaning. Although some sports become arenas for performance optimisation and competition, others are deliberately kept free from datafication to preserve their recreational value. Evidence of this is that when physical activity has been reshaped by digital self‐tracking, performing it without measurement can seem like a waste of time. In such cases, individuals are uncertain about what they *actually* accomplished—because the true measure is only accessible through technology—and also experience a sense of loss, as they miss out on contributing to the statistical capital that each tracked session helps to build.

If I don’t have it [the smartwatch] for a day and I go exercise, I’m like, I don’t know how much I worked out, I don’t know how many calories I burned.

(Victoria)

FAs promote the standardisation and routinisation of users' everyday lives to introduce numeric comparability and competition. As we have shown, using IPs relies on a long process of self‐discipline in managing the disease and operating within highly prestructured contexts—lifestyles already adjusted to diabetes, technical knowledge and institutional support. In contrast, FAs function within unstructured or semi‐structured environments, such as sports practices, subtly shaping these settings over time to introduce the essential frameworks that facilitate comparability, the basis of competition. For example, when asked about how their habits have changed with the use of FAs, these users respond as follows:

Yes, the issue of properly structuring training […] Yes, it really helps with structuring the routine.

(Pablo)

Yes, in fact, it has made me more organised with my meals […] It also allows me to plan my day better, determine the foods I will consume over time, and, well, aesthetically as well, make the difference noticeable.

(Juan)

Other forms of routinisation of everyday life are less evident. Lucas explains how tracking his runs has led him to follow a fixed route:

So, yeah, I’ve got a set route when I go uphill—I know it really well. It goes through certain streets, and I always follow it to the letter because that way, I’ve got a point of comparison.

Changing the route would make it difficult to assess progress, so the tracking device encourages him to run along the same streets, ensuring a consistent basis for comparison and competition.

In the same sense, the effort to translate everyday life into computable data reinforces routines. For instance, Mateo, a user of the weight‐loss application FatSecret, describes how it influences his eating habits as follows:

I’m the kind of person who almost always eats the same thing—same breakfast, etc. You just save it, and it’s basically about adding it.

Because the new breakfast feature requires users to manually enter their food intake into the application, he spontaneously chooses to repeat the same routine every day.

The importance of datafication in the routinisation of habits becomes more evident when it becomes difficult or impossible:

I used to find myself saying no to certain gatherings because, in the end, you’d be drinking alcohol or picking at a platter, and you don’t really know what’s in it—you can’t log it into the app.

(Leo)

The inability to translate certain social or informal eating situations into data leads him to avoid them altogether, privileging contexts that are more easily datafied.

Thus, users of FAs experience two complementary modes of digital mediation—conscious guidance and subtle behavioural modulation—seamlessly embedded in daily life. These strategies discreetly steer decision‐making while gradually shaping behaviour through micro‐management, fostering a sense of coherence that naturalises the human–technology coupling.

This perceived fluency, however, entails a cost: the standardisation of routines to render life legible to digital systems. The supposed continuity between human and technology is not inherent but actively constructed through the imperceptible ways in which FAs condition users' habits, gradually aligning them with data‐driven logics.

In contrast to healthism's emphasis on meaning‐making and reflexivity, FAs prioritise quantification over interpretation, promoting a mode of self‐relation that bypasses discursive engagement in favour of real‐time feedback, gamified incentives and measurable outcomes. FAs externalise self‐care through data and continuous benchmarking against others. Health is no longer framed as a private moral imperative but as a performative achievement within algorithmic systems. Through this shift, FAs extend the logic of responsibilisation into new terrains of surveillance, competition and digital self‐optimisation.

## 6 Making Data Speak: The Discursive Conditions of Digital Truth

The perception of data as intimate and authoritative truths should not be taken for granted. On the one hand, an analysis of how FA use imperceptibly shapes the human–technology relationship reveals that this coupling operates at an infra‐subjective and sub‐semantic level, activating prepersonal, precognitive and preverbal elements (Lazzarato 2014, 31). In this sense, it constitutes a machinic formation that produces a mode of capture which does not interpellate individuals as subjects but incorporates them as functional components within broader sociotechnical configurations (Deleuze and Guattari 1987, 457).

Yet, the production of this experience of continuity is not exhausted at this level. It also entails the shaping of subjects who have internalised specific cultural imaginaries and normative frameworks surrounding the body, health and technology. In other words, data are not inherently meaningful; they must be contextualised and interpreted. To acquire significance, they need to be embedded within narratives, situated in particular contexts and actively mobilised in meaning‐making processes (Dourish and Gómez Cruz 2018).

The importance of the cultural and discursive context that invisibly sustains the coupling between humans and technology becomes most apparent when this continuity is disrupted. In the interviews, such disruptions took two primary forms. The first originates with the user, who engages compulsively with data in pursuit of reassurance, control or self‐affirmation.

For example, IP user Sofía contrasts her experience with diabetes before and after adopting this technology. She explains that previously, she would only measure her glucose levels at specific moments throughout the day, whereas now, she has continuous access to this information. Although she undoubtedly sees this as a positive development, she acknowledges that it can sometimes feel overwhelming:

Many times a day, I have the pump connected to my phone, so sometimes, while I’m already using it, I open the app to check my levels. If I had to estimate, I’d say more than 15, maybe even over 20 times a day. […] I think I unconsciously developed the habit of checking myself. […] I find it reassuring to have numbers, although they can sometimes be overwhelming.

(Sofía)

The compulsive drive to seek out numbers—without any apparent reason other than accumulating more—is dangerous. A certain amount of data may be reassuring, but beyond that threshold, the subject is flooded with information they can no longer interpret or integrate meaningfully. This saturation produces anxiety and desubjectivation: a weakening of the subject's capacity to narrate, symbolise or humanise the data. Lacking a discursive framework to make sense of the numbers, users are left with a stream of raw metrics that impose themselves without mediation, undermining the very autonomy these technologies claim to enhance.

In the second form, the rupture stems from the device itself, whose automated functions produce normative outputs experienced as intrusive, disconnected or misaligned with the user's lived experience. This distinctive agency of digital technologies becomes especially apparent when users do not identify with the subject being hailed by them.

So, it’s like the pump is the one thinking about it all day. And that makes it really hard not to be aware of it. Because, yes, there’s always something going on. I call my pump ‘*the toxic one’ [‘la tóxica’]* because it won’t stop beeping until you press the buttons. This ‘*toxic one’* just won’t let you live in peace.

(Susana)

What is striking about this vignette is that, from the user's perspective, the pump is not addressing her but a generic other. What makes the IP ‘toxic’ is precisely that it imposes a normativity she cannot recognise as her own. In this context, desubjectivation becomes even more radical than in the previously discussed experiences of being overwhelmed. Here, the subject is not only saturated by data she cannot process but also fully excluded from the process of meaning‐making. The device continues to operate and generate normative expectations regardless of whether the user identifies with them. In the same sense, an FA user recounts the following:

The step count or distance isn’t something I pay much attention to, but the watch keeps reminding me automatically. That kind of thing already bothers me because it’s not something I’m interested in. Every so often, the watch pops up with messages like, *‘Let’s start tracking your steps. You’ve taken this many steps, here’s how many you have left.’* Or *‘keep going, you’re doing great!’* … but I don’t remember setting it up, and I can’t disable it either.

This vignette further illustrates that when imposed digital frameworks do not align with the user's lived experience, the result is a sense of alienation from one's self‐tracking data. The digital agency takes on a machinic, non‐negotiable form, which cannot be resisted or reinterpreted but only be physically rejected by silencing the device. Crucially, this is not a matter of the device producing inaccurate information, but of the absence of a discursive framework capable of rendering that information meaningful and legitimate. In this case, the subject is not merely overwhelmed or anxious; she is displaced.

Therefore, these experiences—whether driven by compulsive user engagement or by the device's unsolicited agency—reveal a deeper dynamic at play. DSTPs do not simply collect data; they operate at an infra‐subjective level, capturing granular signals and producing supraindividual numerical categories—the generic other—‘without ever appealing to the subject, without ever calling on them to account for who they are or who they might become’ (Rouvroy and Berns 2013, 174). In this sense, the subject is neither the originator nor the interlocutor of the data‐driven process. Their presence in this coupling depends on how they have learnt to understand their body, interpret digital data and conduct their lives through a numerical matrix.

Crucially, these moments of rupture expose the cultural and discursive scaffolding that ordinarily sustains the experience of coherence, agency and personalised truth. It is precisely when this supporting framework momentarily falters that the underlying logic, as described by Rouvroy and Berns (2013), becomes visible: a regime of algorithmic subjectivation that governs without address, negotiation or justification.

This marks a key difference between DSTPs and predigital healthism. Although both rely on discursive regimes to produce legitimacy and meaning, they relate to this dependency in fundamentally different ways. Healthism openly embraces its discursivity through therapeutic language; it presupposes a subject engaged in the labour of self‐understanding, capable of articulating meaning and narrating their condition. DSTPs, by contrast, reaffirm their authority precisely by disavowing discursivity. They present their outputs as objective, self‐evident and requiring no further interpretation beyond the number itself. In other words, the human–technology suture is enabled by a specific cultural denial of culture itself—an erasure of the mediating role of context, narrative and meaning‐making. In this sense, DSTPs' epistemic legitimacy is not only grounded in the technical capacity of devices to access truth but in broader imaginaries that portray digital data as offering direct, unmediated access to an underlying molecular reality.

## 7 Conclusion

Crawford (1980) coined the term ‘healthism’ to describe an ideology that assigns individuals primary responsibility for managing their health, framing well‐being as the outcome of personal lifestyle choices while intertwining medical and therapeutic discourses. Since then, research has shown how neoliberalism has driven the spread and cultural legitimisation of this ethos (Schrecker and Bambra 2015; Schrecker 2016; Aamann 2020; Spratt 2023; Card and Hepburn 2023). This dynamic is particularly evident in Chile, where decades of neoliberal health governance have entrenched a strongly individualising orientation (Unger et al. 2008; Araujo and Martuccelli 2012; Rotarou and Sakellariou 2017).

Within this setting, scholars argue that digital self‐tracking practices (DSTPs) not only participate in but also intensify this transfer of responsibility (Lupton 2013a, 2013b, 2016; Boka and Pöll 2016; Elias and Gill 2018; Badr 2022; Wieczorek and Rossmaier 2023). Drawing on the cases of insulin pumps (IPs) and fitness applications (FAs) in Chile, this study shows that DSTPs both reinforce and transform this neoliberal logic.

The analysis reveals that the emerging digital subject continues to be held accountable for the conduct of life. However, IPs reinforce this demand within tightly regulated medical frameworks, where users engage with expert knowledge and technological systems while also relying on personal experience, producing hybrid forms of agency that blur boundaries between formal medicine and self‐care. By contrast, FAs encourage self‐optimisation through gamified feedback and routine standardisation, extending healthism into domains of surveillance, competition and behavioural modulation.

Therefore, digital healthism continues to promote the neoliberal ethos of individualisation. While reinforcing this imperative, it simultaneously constrains individual agency by casting doubt on the possibility of accessing personal truth without technological mediation. Unlike its predigital form, which relied on a humanist onto‐epistemology assuming direct access to self‐knowledge, DSTPs derive legitimacy from a posthumanist view of the body and self at molecular and numerical scales, accessible only through technological interfaces.

This reveals a crucial paradox: DSTPs legitimise themselves through discourses that deny the very power of discourse, elevating numbers as the most reliable source of truth. Although predigital healthism embraced therapeutic language and narrative as means of self‐understanding, DSTPs erase the mediating role of context and meaning, presenting digital data as providing unmediated access to reality. Within this configuration, the subject of digital healthism is suspended between the enduring demand to ‘know thyself’ and the recognition that such knowledge depends on the very technologies that both empower and constrain it.

## 8 Strengths, Limitations and Future Directions

The comparative design of this study constitutes a key contribution, allowing for a nuanced exploration of how DSTPs operate across institutionalised medical settings and everyday wellness cultures. The analysis advances current debates on the reconfiguration of subjectivity through digital health technologies by integrating empirical data with theoretical perspectives from critical sociology and posthumanism.

However, this study has several limitations. Although the sample was socioeconomically diverse, it was relatively small and geographically concentrated in urban Chile, which may limit the generalisability of the findings. Moreover, the retrospective nature of the data obtained through semi‐structured interviews restricts the ability to capture the longitudinal evolution of users' relationships with DSTPs from a prospective perspective. Finally, the focus on insulin pumps and fitness applications excludes other relevant technologies, such as reproductive tracking tools, mental health applications or emerging biosensors.

Therefore, future research could build on these findings by employing longitudinal and ethnographic approaches that track how digital self‐tracking unfolds over time and shapes subjectivity. Comparative studies across diverse cultural and healthcare systems would further enhance understanding. Finally, examining practices of resistance, refusal or selective disengagement from DSTPs could shed light on alternative forms of agency that move beyond responsibilisation and self‐optimisation.

## Author Contributions

**Rodrigo De La Fabián:** conceptualisation, methodology, formal analysis, writing – original draft, writing – review and editing. **Alvaro Jiménez‐Molina:** methodology, formal analysis, writing – original draft, writing – review and editing. **Francisco Pizarro Obaid:** formal analysis, writing – original draft, writing – review and editing. **Jimena Carrasco Madariaga:** formal analysis, writing – original draft, writing – review and editing.

## Ethics Statement

This study was approved by the Research Ethics Committee (Comité de Ética en Investigación, CEI‐UDP) of Universidad Diego Portales (approval number 17‐2024). All participants provided written informed consent for using anonymised data for scientific publication.

## Consent

All participants provided written informed consent, including permission to use anonymised excerpts from their interviews for scientific publication.

## Conflicts of Interest

The authors declare no conflicts of interest.

## Permission to Reproduce Material From Other Sources

The authors have nothing to report.

## Data Availability

The data supporting this study's findings consist of qualitative interview transcripts containing sensitive and potentially identifying information. Because of ethical and privacy considerations, these data are not publicly available. Requests for access to anonymised excerpts may be considered case by case and should be directed to the corresponding author.

## Endnotes

^1^
Lupton (2014b) identifies five modes of self‐tracking: (1) *private self‐tracking*, where individuals monitor aspects of their health or well‐being on their own initiative, outside institutional settings; (2) *pushed self‐tracking*, which is encouraged by medical or technological actors but still involves active user engagement; (3) *communal self‐tracking*, which entails sharing personal data with others for mutual learning or collective goals, though often still framed by individualistic values; (4) *imposed self‐tracking*, which is mandated by institutions (such as employers, schools or legal authorities), with limited user agency; and (5) *exploited self‐tracking*, where data—regardless of the user's initial consent—are appropriated by third parties (e.g., corporations) for commercial or surveillance purposes. For analytical purposes, we group these modes into two broad categories: The first three represent voluntary practices, whereas the last two correspond to nonvoluntary or externally driven forms of self‐tracking. This article focuses on the first two modes, *private* and *pushed self‐tracking*, as developed in the main text.

^2^
Under the Ricarte Soto Law, users are provided with insulin pumps manufactured by Medtronic. The device model is updated every 4 years. At the time of the interviews, participants were using either the MiniMed 670 g or the MiniMed 780 g. Both models include the following components: (1) The pump unit stores insulin and regulates its delivery according to programmed settings; (2) the infusion set, consisting of a thin cannula inserted subcutaneously and connected to the pump via tubing, allowing for continuous insulin infusion; (3) the continuous glucose monitor (CGM), which measures glucose levels in real time via a sensor placed under the skin. In these models, the CGM is integrated with the pump, enabling automated adjustments to insulin delivery; (4) a user interface, typically a digital screen on the pump itself, which allows the user to monitor data, receive alerts and manually adjust settings when needed.

^3^
The MiniMed system is a programmable insulin pump that delivers continuous subcutaneous insulin. Meanwhile, the FreeStyle Libre 1 is a flash glucose monitor that allows users to scan a sensor to access real‐time glucose data, without active insulin delivery.

^4^
Finger‐pricking devices provide an immediate and direct measurement of blood glucose levels, offering a real‐time snapshot of circulating glucose. In contrast, continuous glucose monitors (CGMs) measure glucose in the interstitial fluid with a slight delay. However, unlike finger‐pricking devices, CGMs can detect trends over time, indicating whether glucose levels are rising or falling, allowing for better anticipation and management of fluctuations.
